# Characterization of a pESI-like plasmid and analysis of multidrug-resistant *Salmonella enterica* Infantis isolates in England and Wales

**DOI:** 10.1099/mgen.0.000658

**Published:** 2021-10-14

**Authors:** Winnie W. Y. Lee, Jennifer Mattock, David R. Greig, Gemma C. Langridge, David Baker, Samuel Bloomfield, Alison E. Mather, John R. Wain, Andrew M. Edwards, Hassan Hartman, Timothy J. Dallman, Marie A. Chattaway, Satheesh Nair

**Affiliations:** ^1^​ Gastrointestinal Bacterial Reference Unit, Public Health England, London, UK; ^2^​ MRC Centre for Molecular Bacteriology and Infection, Imperial College London, London, UK; ^3^​ Norwich Medical School, University of East Anglia, Norwich, UK; ^4^​ Roslin Institute and Royal School of Veterinary Studies, University of Edinburgh, Edinburgh, UK; ^5^​ Microbes in the Food Chain, Quadram Institute Bioscience, Norwich, UK; ^6^​ University of East Anglia, Norwich, UK

**Keywords:** *bla*_CTX-M-65_, ESBL, MDR, pESI and pESI-like plasmids, *S*. Infantis

## Abstract

*
Salmonella enterica* serovar Infantis is the fifth most common *Salmonella* serovar isolated in England and Wales. Epidemiological, genotyping and antimicrobial-resistance data for *S *. *enterica* Infantis isolates were used to analyse English and Welsh demographics over a 5 year period. Travel cases associated with *S *. *enterica* Infantis were mainly from Asia, followed by cases from Europe and North America. Since 2000, increasing numbers of *S *. *enterica* Infantis had multidrug resistance determinants harboured on a large plasmid termed ‘plasmid of emerging *S *. *enterica* Infantis’ (pESI). Between 2013 and 2018, 42 *S *. *enterica* Infantis isolates were isolated from humans and food that harboured resistance determinants to multiple antimicrobial classes present on a pESI-like plasmid, including extended-spectrum β-lactamases (ESBLs; *bla*_CTX-M-65_). Nanopore sequencing of an ESBL-producing human *S *. *enterica* Infantis isolate indicated the presence of two regions on an IncFIB pESI-like plasmid harbouring multiple resistance genes. Phylogenetic analysis of the English and Welsh *S *. *enterica* Infantis population indicated that the majority of multidrug-resistant isolates harbouring the pESI-like plasmid belonged to a single clade maintained within the population. The *bla*_CTX-M-65_ ESBL isolates first isolated in 2013 comprise a lineage within this clade, which was mainly associated with South America. Our data, therefore, show the emergence of a stable resistant clone that has been in circulation for some time in the human population in England and Wales, highlighting the necessity of monitoring resistance in this serovar.

## Data Summary

Public Health England (PHE) and Quadram Institute Bioscience Illumina fastq files are available from the National Center for Biotechnology Information (NCBI) BioProject PRJNA248792. All Illumina accession numbers are given in Table S1 (available with the online version of this article), including the Illumina equivalents of the Nanopore sequenced samples below.

Nanopore fastq files for *Salmonella enterica* Infantis isolate 114061 (BioSample SAMN08732049) are available from BioProject PRJNA248792 under Sequence Read Archive (SRA) accession number SRR13518322. The corrected 114061 assembly can be found under BioProject PRJNA315192 under the following accession numbers: chromosome, CP070302; pESI, CP070303.

Nanopore fastq files for *S *. *enterica* Infantis isolate 91264 (BioSample SAMN03478587) are available from BioProject PRJNA248792 under SRA accession number SRR13674758. The corrected 91264 assembly can be found under BioProject PRJNA315192 under accession number CP070301 (chromosome).

Supplementary Material can be found on FigShare (at https://doi.org/10.6084/m9.figshare.14815824).

**Impact Statement**

Whole-genome sequencing (WGS) has revolutionized public-health microbiology and has been used routinely at Public Health England since April 2015 for identification, epidemiological surveillance and monitoring antimicrobial resistance (AMR). Analysing WGS data allows the *in silico* interrogation of emerging resistance determinants, aids in investigating mechanisms conferring resistance and enables characterization of drug-resistance regions. Integration of WGS data with epidemiological information supports tracking of transmission and AMR in the *Salmonella* population and, hence, assists in assessing the risk to public health. In this study, *in silico* gene detection and phylogenetic analysis identified the presence of multidrug-resistant *Salmonella enterica* Infantis isolates with extended-spectrum β-lactamase AMR determinants harboured on a megaplasmid (pESI-like plasmid) in the English and Welsh human population. This pESI-like plasmid seems to be maintained in the human population and has been reported both in human and poultry populations in many regions of the world. Understanding AMR carriage and transmission patterns provides information to support appropriate clinical management and inform implementation of public-health control measures.

## Introduction

Non-typhoidal *Salmonella* infections are generally self-limiting (but may cause disease requiring antimicrobial treatment), cause an estimated 93.8 million global cases per annum, with approximately 86 % of these being foodborne gastroenteritis cases [1]. The prevalence of various serovars differs across the world, with *Salmonella enterica* serovar Infantis emerging as the fourth most common serovar in humans across Europe [2]. The population structure of *S *. *enterica* Infantis isolates in England and Wales consists of two eBurst groups (eBGs), eBG31 and eBG297, with eBG31 being the predominant group [3]. This serovar is also dominant in animal reservoirs, especially in poultry where it accounted for 36.7 % of isolates in Europe in 2018 [4–6]. Widespread use of antimicrobials in the animal production industry contributes to antimicrobial resistance (AMR) and the dissemination of *S *. *enterica* Infantis into the food chain, with transmission to humans a growing public-health concern [7, 8].

The increasing incidence of *S *. *enterica* Infantis may be complicated by the acquisition of resistance to medically important antimicrobials. Many resistance determinants are harboured on mobile genetic elements such as plasmids that are found and circulate within the bacterial kingdom. Emerging *S *. *enterica* Infantis isolates harbouring plasmids carrying multiple resistance determinants have been reported recently, with one of the first cases being reported in Israel [9]. The plasmid described, ∼280 kbp in size, contained genes conferring resistance to tetracycline, trimethoprim, sulfamethoxazole, antiseptics and heavy metals, and was termed 'plasmid of emerging *S *. *enterica* Infantis' (pESI). Furthermore, this plasmid contained genes associated with virulence, including those encoding yersiniabactin (*ybt* operon) and fimbriae (*fea* and *ipf* operons), which enhance the colonization capability and fitness of the bacterium [9]. Similar pESI-like plasmids that have evolved to confer resistance to extended-spectrum β-lactams via the acquisition of *bla*_CTX-M-1_ and *bla*_CTX-M-65_ genes have been reported across the globe, including the United States, South America and Europe [4–11].

Whole-genome sequencing (WGS) has been used by Public Health England (PHE) since April 2014 for supporting outbreak investigations, studying *Salmonella* population structures and understanding *Salmonella* pathogenicity [3, 12]. Characterization of *Salmonella* genomes with WGS using bioinformatics provides the ability to screen and differentiate the resistance genes present, as well as characterize drug-resistance regions, which cannot be carried out with phenotypic antimicrobial-susceptibility tests [13, 14].

Although recent studies have described the emergence of multiple-drug resistant (MDR) and extended-spectrum β-lactamase (ESBL)-producing *S *. *enterica* Infantis in multiple countries, it has not been well described in England and Wales [8, 15]. Therefore, the aim of this study was to use passive surveillance and WGS to investigate the epidemiology, population structure and AMR of current and historical *S *. *enterica* Infantis isolates from humans and poultry-related food sources in England and Wales.

## Methods

### Bacterial isolates and datasets

*S *. *enterica* Infantis isolates (*n*=624) from eBG31 collected by the Gastrointestinal Bacterial Reference Unit (GBRU), PHE, from local and regional hospitals in England and Wales between 13.09.2013 and 14.11.2018 were sequenced at PHE in this study (Table S1). To ascertain whether the MDR isolates had been circulating in the human population and whether there was a potential historical source, a further 171 historical isolates (prior to the implementation of WGS) from the GBRU culture stores, isolated between 2000 and 2013, were also sequenced at Quadram Institute Bioscience. Human isolates were sourced from urine, blood or from cases with a history of foreign travel, and food isolates associated with poultry were given preference. Phylogenetic analysis, AMR analysis and pESI-like plasmid detection were carried out on these 795 genomes (Table S1). Thirty isolates predicted to have ESBL determinants were subjected to antimicrobial-susceptibility testing (Table S2). The dataset selected for the demographic study included 943 *S *. *enterica* Infantis isolates that were sequenced at PHE (of which 528 isolates were used for the phylogenetic analysis), derived solely from human cases between January 2014 to December 2018 (Table S3).

### Demographics

Demographic data for all the 943 human *S *. *enterica* Infantis isolates from England and Wales (Table S3) stored in the PHE’s in-house Gastro Data Warehouse (GDW) were obtained. Data containing age, gender, geography, date of isolation, travel history, serovar identification and antimicrobial susceptibility were extracted for analysis. WGS data were also available for these isolates for *in silico* AMR typing (Table S2). Ethical approval was not required for this study as PHE is permitted to process these data for surveillance purposes, outlined in the National Health Service Act 2006.

### Antimicrobial-susceptibility testing

Minimum inhibitory concentrations (MICs) were available for 30 isolates exhibiting ESBL genotypes (Table S2). These were determined by agar dilution using Mueller–Hinton agar for the standard panel of antibiotics recommended by the European Committee on Antimicrobial Susceptibility Testing (EUCAST). EUCAST breakpoints and screening concentration criteria were used for interpretation (https://eucast.org/clinical_breakpoints/). Confirmation of azithromycin MIC was performed by EtestVR (bioMérieux). Temocillin and cefoxitin were included in the panel to aid detection of OXA-48-like carbapenemases and AmpC production, respectively. ESBL detection was confirmed using aztreonam, cefotaxime/cefotaxime+clavulanic acid (4 µg ml^−1^), ceftazidime/ceftazidime+clavulanic acid (4 µg ml^−1^) and cefepime/cefepime+clavulanic acid (4 µg ml^−1^)

### Illumina sequencing and data processing

Genomic DNA extraction of *S *. *enterica* Infantis isolates was carried out using a modified protocol of the QIAsymphony DSP DNA midi kit (Qiagen) as described by Nair *et al*. [14]. Extracted genomic DNA was then prepared using the NexteraXT method and sequenced with a standard 2×101 bp FAST protocol on a HiSeq 2500 instrument (Illumina). Trimmomatic (v0.27) was used to trim sequence data with leading and trailing at <Q30 [16]. The 171 historical *S *. *enterica* Infantis isolates were sequenced at Quadram Institute Bioscience on the Illumina NextSeq platform, following methods described by Rasheed *et al*. [17], demultiplexed on climb [18] and trimmed using Trimmomatic (v0.36).

### Genotyping

Sequence type (ST) and serovar were determined from the reads using most (v1.0) as previously described by Tewolde *et al*. [19] and eBG as described by Achtman *et al*. [20]. PlasmidFinder (v2.1) (http://cge.cbs.dtu.dk/services/PlasmidFinder/) was used to detect the presence of known replicon types of plasmids in the isolates studied [21].

### *In silico* AMR typing

Resistance genes for the 943 human isolates used for the demographic study were identified using GeneFinder (https://github.com/phe-bioinformatics/gene_finder), a customized algorithm that uses Bowtie2 (v2.3.5.1) [22] to align reads to a set of reference sequences and SAMtools (v1.8) [23] to generate an mpileup file, as previously described by Day *et al*. [24]. Briefly, the data are parsed based on read coverage of the query sequence (100%), consensus base-call on variation (>85 %) and the nucleotide identity (>90 %) to determine the presence of the reference sequence or nucleotide variation within that sequence. β-Lactamase variants were determined with 100 % identity using the reference sequences downloaded from ResFinder (https://cge.cbs.dtu.dk/services/ResFinder-4.0/) or National Center for Biotechnology Information (NCBI) (https://www.ncbi.nlm.nih.gov/pathogens/beta-lactamase-data-resources) β-lactamase data resources. Known acquired resistance genes and resistance-conferring mutations relevant to β-lactams, fluoroquinolones, aminoglycosides, chloramphenicol, macrolides, sulfonamides, tetracyclines, trimethoprim, rifamycins and fosfomycin were included in the analysis [25, 26] (Table S2).

*In silico* AMR typing was also performed using ariba (v2.10.1) on the 624 *S *. *enterica* Infantis isolates and the 171 historical *S *. *enterica* Infantis isolates used for the WGS analyses [27]. *aac(6’)-Iaa* was excluded from all calculations as it is known to be a cryptic resistance gene (Tables S2 and S4).

### Nanopore sequencing and data processing

Nanopore sequencing was performed on two occasions, the first was to sequence isolate 91264 (SRR1968494) to act as a reference in the reconstruction of a phylogenetic tree for eBG31. The second instance was to sequence human-derived isolate 114061 (SRR6854755), to characterize the drug resistance determinants as it exhibited extended-spectrum β-lactam and multidrug resistance, as well as the presence of an IncFIB plasmid (Tables S1 and S3).

Both isolates 114061 (SRR6854755) and 91264 (SRR1968494) were cultured and genomic DNA extracted using a Fire Monkey kit (RevoluGen) following the manufacturer’s instructions except for the incubation period with lysozyme, which was increased to 1 h. Library preparation was performed using the Rapid Barcoding kit SQK-RBK004 (Oxford Nanopore Technologies). The prepared library was loaded on a FLO-MIN106 R9.4.1 flow cell (Oxford Nanopore Technologies) and sequenced on the MinION system (Oxford Nanopore Technologies) for 48 h using MinKNOW software (v1.18).

Isolate 91264 (SRR1968494) was basecalled using Albacore (v2.0.1) (Oxford Nanopore Technologies). The fastq output was assembled using Canu (v1.7) with default parameters but for useGrid=false [28]. The assembly was then polished using the Nanopolish methylation dcm option, flag min-candidate-frequency 0.5 using bwa (v0.7.17) [29] and SAMtools (v1.8), followed by polishing using Pilon (v1.22) and Racon (v1.3.2) [30, 31] with the Illumina-generated reads for isolate 91264 (SRR1968494).

Isolate 114061 (SRR6854755) was basecalled using Albacore (v2.1) (Oxford Nanopore Technologies), converted to fastq format and then demultiplexed. The reads (fastq) were then trimmed using Porechop v0.2.0 [32], removing sequencing adapters and barcodes. Reads (fastq) were then filtered using Filtlong (v0.1.1) [33], the highest quality and longest reads (worth 50× coverage of the average *Salmonella* genome) were taken for further processing.

The filtered and trimmed fastq reads were then assembled using Flye (v2.6) [34] with the --plasmids parameter enabled. Correction of the assembly of the isolate was conducted in concordance to Yara *et al*. [35]. The assemblies were corrected in a three-step process. Nanopolish (v0.10.2) [36] was utilized for trimmed Oxford Nanopore Technology (ONT) fastq and fast5 reads generated for each sample using --fix-homopolymer, --methylation-aware=dcm,dam and --min-candidate-frequency=0.1. Secondly, Pilon (v1.22) [31] was used with Illumina fastq reads as the query dataset with the use of bwa (v0.7.17) and SAMtools (v1.7) on the assembly. Finally, Racon (v1.2.1) also using bwa (v0.7.17) and SAMtools (v1.7) was used with the Illumina reads for two cycles to produce a final assembly.

### Characterization of the region encoding β-lactam and other resistance determinants

The assembled *S *. *enterica* Infantis isolate (114061) (SRR6854755) genome was visualized with Bandage (v.0.6.2) [37] (Fig. S1). blastn (v5) (blast.ncbi.nlm.nih.gov/Blast.cgi) was used to detect AMR genes, in particular genes associated with ESBL-producing *S *. *enterica* Infantis isolates as previously described such as *bla*_CTX-M-65_, *fos-3A*, *tetA*, *dfrA14*, *sul1* and *floR*, and their location in the assembly graph [5]. Prokka (v1.12) was used to annotate genome sequences (http://www.ncbi.nlm.nih.gov/pubmed/24642063) [38] and Artemis (v18.00) (www. sanger.ac.uk/tool/artemis) was used to visualize the resistance regions [39]. The orientation and position of specific genes were drawn using Easyfig (v2.1) [40].

brig (v0.95) [41] was used with blast+ (https://blast.ncbi.nlm.nih.gov/Blast.cgi) parameters of 95 % sequence similarity and an *E* value of 1×10^–10^ to compare the IncFIB pESI plasmid isolated from an ESBL-producing *S *. *enterica* Infantis in chickens from the United States (CP016409.1) and the pESI plasmid from the *S *. *enterica* Infantis found in Israel (ASRF01000000.1) to the UK pESI plasmid (CP070303.1) from sample 114061 (SRR6854755) (of human origin) in this study. The output image displays the degree of similarity between the ring of reference in the centre and the genome of the isolates used.

### Identification of pESI presence

Seven hundred and ninety-five *S *. *enterica* Infantis isolates (including the 171 historical isolates) were analysed (Table S1). The presence of pESI in each of the genomes was determined using a pESI pseudomolecule that was created with the long-read sequenced eBG31 reference genome and the pESI contigs from the whole-genome assembly of an Israeli human *S *. *enterica* Infantis (ASRF01000099–ASRF01000108). The sequences were mapped against this pseudomolecule using smalt (v0.7.6) (seed=5); coverage was then determined using SAMtools (v1.8) [23]. Heatmaps of pESI coverage were generated, with a read depth of 20 signifying presence, using R (v3.5) with the packages data.table (v1.11.8), phytools (v0.6–63) and ape (v5.2) [42–45]. The heatmaps were analysed to determine which isolates contained pESI. Absence of the plasmid was defined as a read depth below 20 in over 50 % of the plasmid (Table S1).

The presence of pESI-like plasmid in the 795 *S *. *enterica* Infantis isolates used for the WGS analysis was also confirmed by checking for the presence of the *ybtQ* (yersiniabactin) and *ybtX* (yersiniabactin), *feaG* (fimbriae) and *ipfC* (fimbriae) using GeneFinder (https://github.com/phe-bioinformatics/gene_finder) (Table S1). Bandage was utilized to visualize the presence of the *ybtQ*, *feag* and *ipfC* genes on the pESI-like plasmid from *S *. *enterica* Infantis isolate 114061 (SRR6854755) (Fig. S2).

### Phylogenetic analysis

Phylogenetic analysis of the 795 *S *. *enterica* Infantis isolates was carried out as follows. The corrected assembly of isolate 91264 (SRR1968494) was used as a reference genome for the eBG31 SnapperDB (v1.0.6) [46]. Illumina fastq reads of the *S *. *enterica* Infantis genomes were mapped to isolate 91264 using bwa mem (v0.7.12) [29]. Variant positions were identified by gatk (v3.8.0) Unified Genotyper [47] that passed the following parameters: >90 % consensus, minimum read depth of 10, mapping quality (MQ) ≥30. Any variants that remained were imported in the eBG31 SnapperDB (v1.0.6). The eBG31 SnapperDB (v1.0.6) was used to generate a soft-core SNP alignment (>80 % consensus) masking recombination, which was detected by Gubbins (v.2.3.1), and prophages were detected with phaster [48, 49], also any positions where reads could not re-align to self were masked. RAxML (v8.2.12) was used with the nucleotide substitution model GTRCAT, to reconstruct a maximum-likelihood phylogenetic tree that was rooted to its most ancestral node [50].

The phylogeny was annotated with pESI and *bla*_CTX-M-65_ presence using iTOL [51]. The *bla*_CTX-M-65_ clade was extracted using FigTree (v1.4.3) and the accompanying figure (genes present/absent plotted against phylogeny) generated using Phandango [52, 53]. Pairwise SNP distances were calculated using mega7 (version 7180411-i3860) [54].

## Results

### Demographics

Between January 2014 and December 2018, PHE received 42 422 human *Salmonella* isolates from England and Wales, where *S *. *enterica* Infantis (*n*=1093, 2.6%) was the fifth most common serovar identified. Out of the 943 *S *. *enterica* Infantis isolates analysed for the demographic study, 917 were from eBG31 and 26 belonged to eBG297 (Table S2).

**Fig. 1.**
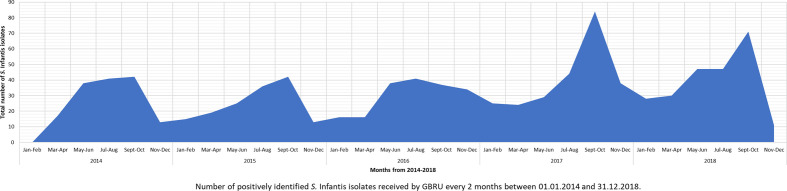
Isolation pattern (number of isolates) of *S *. *enterica* Infantis isolated at GBRU, PHE, between 01.01.2014 and 31.12.2018.

Of the 943 *S *. *enterica* Infantis isolates in this study from patients in England and Wales, 454 (48.1 %) were isolated from females, 487 (51.6 %) were from males and 2 (0.2%) had no gender information (Table S3). The incidence of *S *. *enterica* Infantis infection increased over the study period: in both 2014 and 2015, 150 isolates were reported; which increased to 179, 244 and 218 in 2016, 2017 and 2018, respectively. Submission of *S *. *enterica* Infantis isolates to PHE peaked between July and October for all years studied (Fig. 1, Table S3).

### Travel-associated *S *. *enterica* Infantis cases

**Fig. 2.**
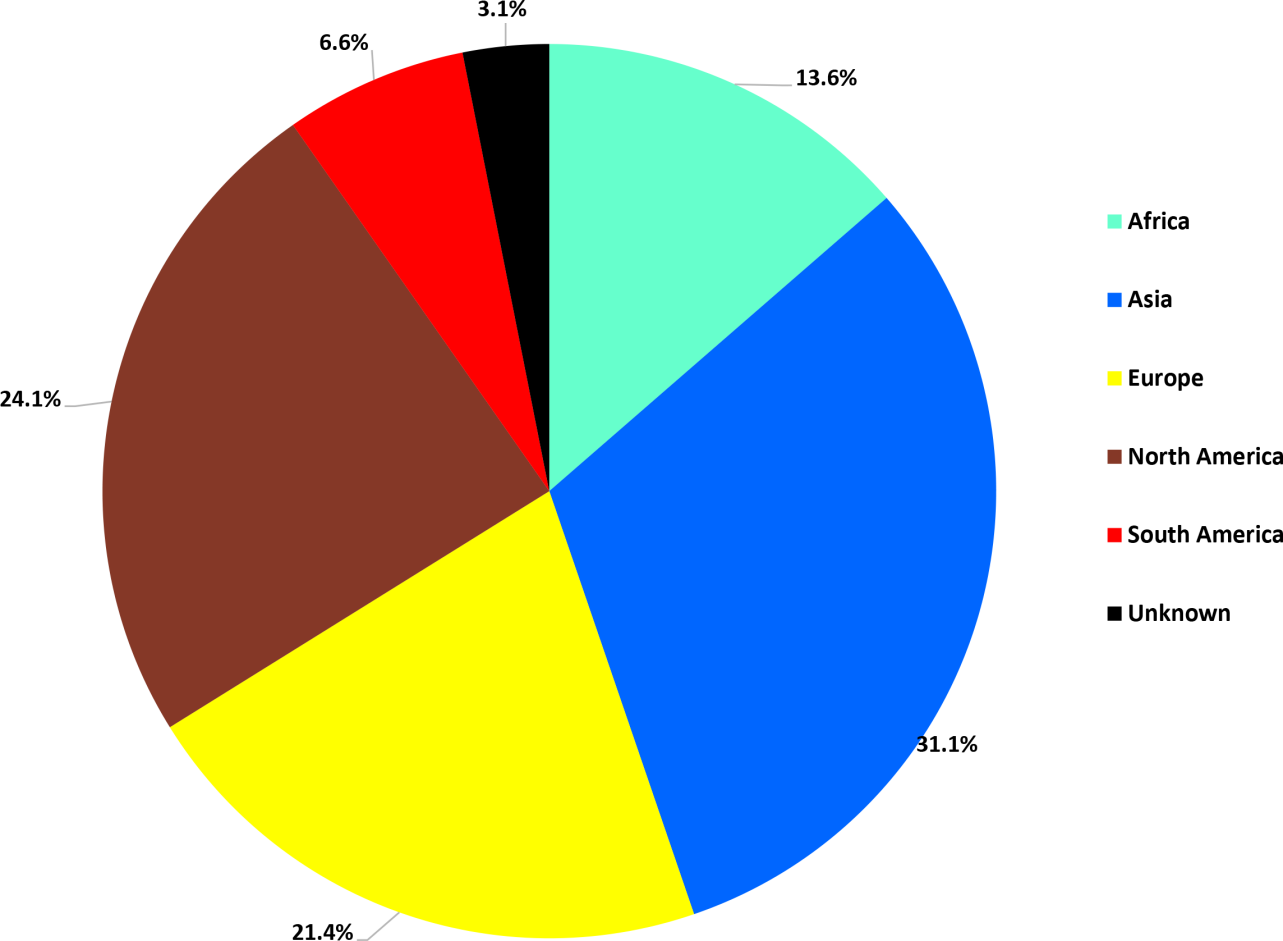
Percentage of *S *. *enterica* Infantis isolates associated with travel between 2014 and 2018 in England and Wales.

Overall, 26.9 % of *S *. *enterica* Infantis isolates were isolated from patients with travel history. Travel cases associated with *S *. *enterica* Infantis were mainly to Asia (31.1%), followed by North America (24.1%) and Europe (21.4%) (Fig. 2).

### *In silico* AMR detection

Genomes of 943 *S *. *enterica* Infantis human isolates used for the demographic study, and the 795 *S *. *enterica* Infantis (human and food) isolates for the phylogenetic analyses, were screened for known genes and point mutations associated with AMR, including ESBL resistance in *Enterobacteriaceae* (Tables S2 and S4). The presence of *bla*_CTX-M-65_, amongst other resistance determinants conferring resistance to β-lactams, aminoglycosides, quinolones, tetracycline, trimethoprim, sulfonamide, chloramphenicol and fosfomycin, was identified in 42 *S *. *enterica* Infantis genomes (including 2 historical isolates – SRR14774811 and SRR14774768 (Table S2). There were 35 human isolates and 7 were from food related to chicken produce (Table S2). Of the human isolates with known travel history, 40.0 % (14/35) were associated with travel to South America, while out of the 7 food products, 6 originated from South America (85.7%). The main resistance profile observed in 25/42 isolates (59.5%) was β-lactam (*bla*_CTX-M-65_), aminoglycosides [*aph(4)-Ia*, *aadD*, *aac(3)-Iva*], fluoroquinolones [*gyrA*(87:D-Y)], trimethoprim (*dfrA-14*), tetracycline [*tet(A)−1*], sulfonamides (*sul-1*), chloramphenicol (*floR*), fosfomycin (*fosA-3*) (AMR profile 1); followed by 12/42 isolates (28.6%) with the same AMR profile except for the absence of the *fosA* gene (AMR profile 2). Two out of forty-two (4.8 %) isolates did not have the *dfrA-14* and *fosA* genes (AMR profile 3). One isolate was negative for the *tet(A)−1* and *sul-1* genes (AMR profile 4). One isolate did not have the *dfrA-14* gene (AMR profile 5), and another was missing the *floR* gene (AMR profile 6) (Table S2). All the isolates (*n*=42) also had genes related to heavy metal resistance, *me*r (mercury) and *ars* (arsenic). Phenotypic antimicrobial-susceptibility testing was performed on 30 of the isolates, which confirmed the multidrug-resistance phenotypes predicted from WGS data (Table S2).

### Location and characterization of AMR determinant loci

**Fig. 3.**
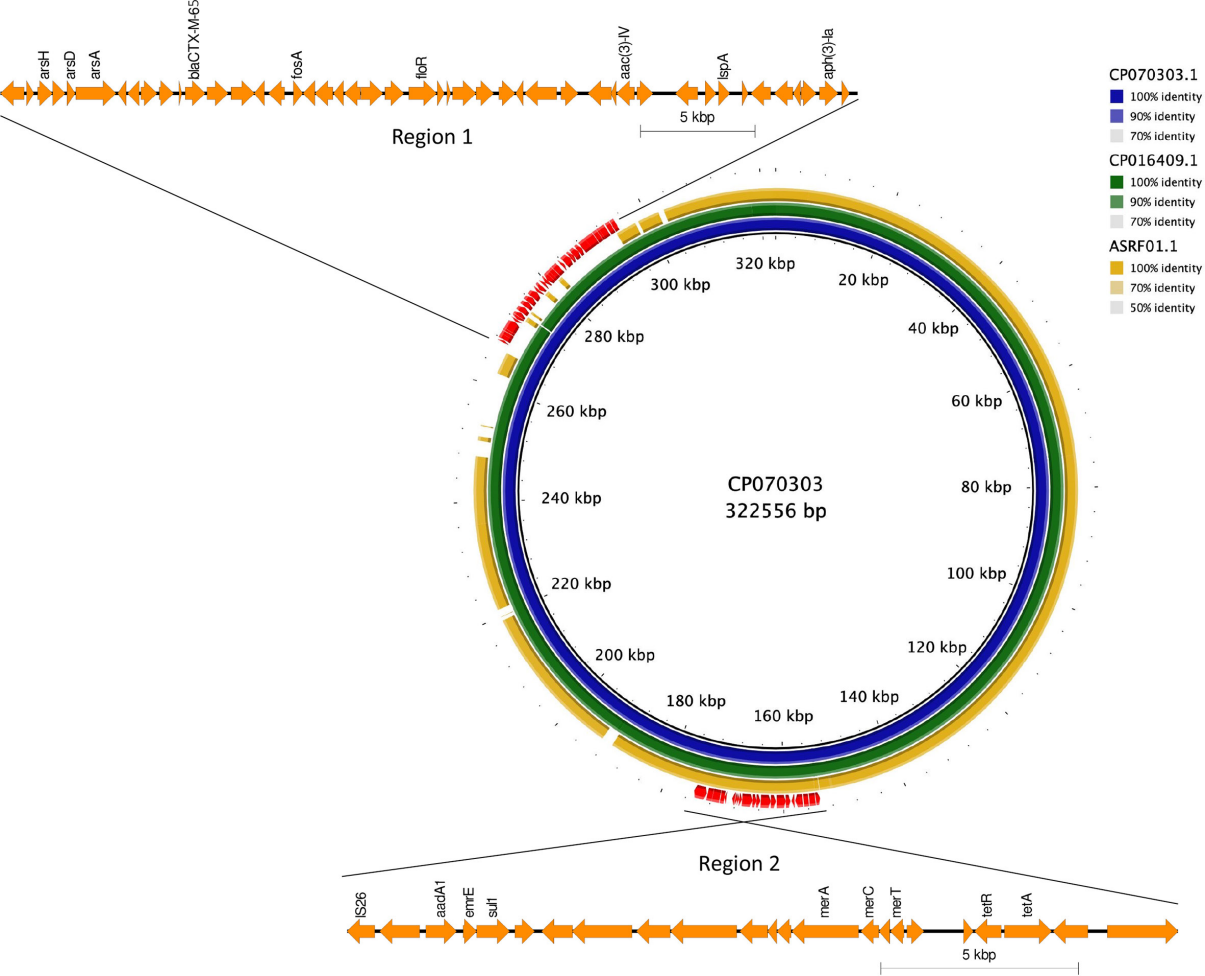
blast comparison of IncFIB plasmid identified in *S *. *enterica* Infantis isolate 114061 (SRR6854755) from a human in this study (inner blue ring ring) compared to a known IncFIB plasmid (NCBI accession no. CP016409) found in *S *. *enterica* Infantis isolated from chickens in the USA (middle green ring) [5] and an *S *. *enterica* Infantis human isolate from Israel (outer yellow ring) [9]. Region 1 consists of the following resistance determinants*: bla*_CTX-M-65_ (ESBL); *fosA3* (fosfomycin); *floR* (chloramphenicol); *aph(4)-la*, *aac(3)-IV*, *aph(3)-la* (aminoglycosides); *dfrA-14* (trimethoprim); *ars* (arsenic). Region 2 confers the following resistance: *tetA* (tetracycline), *sul1* (sulfonamides), *aadA1* (aminoglycosides), *mer* (mercury).

Nanopore sequencing was carried out to investigate the genomic context of the resistance genes in a human-derived *S *. *enterica* Infantis isolate, 114061 (SRR6854755). Isolate 114061 had AMR profile 1 and harboured resistance to all eight antimicrobial classes described in the study (Table S2). Analyses of the 114061 genome indicated that the resistance genes and insertion elements (IS26 and *tnp*) were located in two resistance loci (region 1 and region 2) on a 320 kbp plasmid that showed 99 % sequence similarity to an IncFIB plasmid (NCBI accession no. CP016409) (Figs 3 and S1). Region 2 was approximately 21.0 kbp and included resistance genes for sulfonamides (*sul-1*), aminoglycosides [*aph(4)-Ia*, *aph(3)-la*, *aac(3)-Iva*], tetracycline [*tet(A)−1*] and mercury (*mer*). Region 1 (approximately 36.3 kbp) consisted of the *bla*_CTX-M-65_ gene conferring resistance to extended-spectrum β-lactam drugs, fosfomycin (*fosA-3*), chloramphenicol (*floR*) and aminoglycosides (*aadA1*), as well as the heavy metal arsenic (*ars*) (Table S2).

### Pairwise blastn comparison of IncFIB plasmid harbouring β-lactam resistance

The IncFIB plasmid identified in *S *. *enterica* Infantis isolate 114061 (SRR6854755) from a human in this study was compared to a known IncFIB plasmid (NCBI accession no. CP016409) found in *S *. *enterica* Infantis isolated from chickens in the USA [5] and the original pESI plasmid (NCBI accession no. ASRF01000000.1) isolated from a human, detected in Israel [9]. The brig plot (Fig. 3) showed 99 % similarity between the pESI-like plasmids from the UK human isolate and the chicken isolate, with both isolates having identical resistance regions (regions 1 and 2) (Fig. 3). There was approximately 70 % backbone similarity between the pESI plasmid from the Israeli isolate with the other two plasmids, with the Israeli plasmid lacking resistance region 1 (Fig. 3).

### Presence of pESI-like plasmid, detection of ESBL resistance determinants and phylogenetics analysis of *S *. *enterica* Infantis in England and Wales

**Fig. 4.**
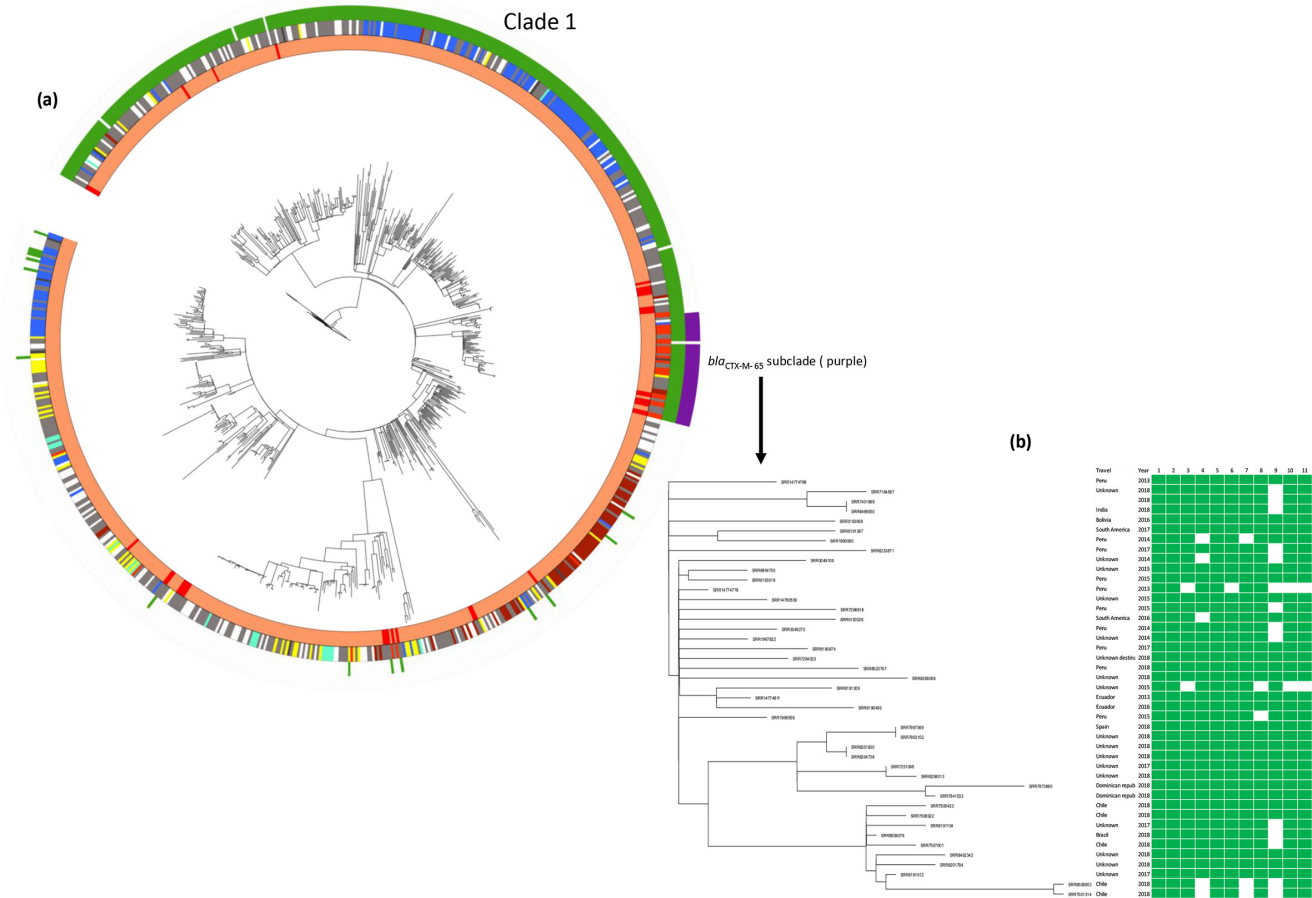
(**a**) Maximum-likelihood phylogeny of 795 *S *. *enterica* Infantis isolates. Clade 1 represents the 368 isolates with pESI-like plasmids. Forty-two isolates harbouring *bla*_CTX-M-65_ fell into a sub-clade (purple). Another isolate (hPHE_159) that fell into this cluster did not harbour the *bla*_CTX-M-65_ gene. Inner ring – isolation source: human (orange), food (red), animal (gold), other (brown) and unknown (grey). Second ring – association with foreign travel: Asia (blue), Africa (green), Europe (yellow), North America (brown), South America (red), unknown destination (black), unknown (grey) and no travel (white). Third ring – pESI-like plasmid presence. Outer ring – isolates carrying *bla*_CTX-M-65_ on a pESI-like plasmid. (**b**) Heat map showing the presence/absence of AMR genes in the 43 isolates that fell into the *bla*_CTX-M-65_ subclade in (**a**). Only isolate hPHE_159 did not harbour the *bla*_CTX-M-65_ gene. 1, *aac(3)IV*; 2, *aac(6)Iaa*; 3, *aadA*; 4, *aph(3')-Ia*; 5, *aph(4)-Ia*; 6, *bla*_CTX-M-65_; 7, *dfrA14*; 8, *floR*; 9, *fosA3*; 10, *sul1*; 11, *tet(A*).

Forty-eight per cent (383/795) of the isolates in the dominant eBG in *S *. *enterica* Infantis, eBG31 (including historical GBRU isolates), harboured a pESI-like plasmid, with the first being seen as early as 2000 (Table S1). Phylogenetic analysis of the eBG31 population showed that 368 (96 %) pESI-positive isolates were present in monophyletic clade 1 and all harboured the IncFIB plasmid (Fig. 4a). The 15 isolates that were pESI positive but fell outside of clade 1 harboured plasmids of different incompatibility groups (8 isolates with IncI1 plasmid, 6 with IncP plasmid and 1 with an IncA/C plasmid). pESI-like plasmids were present in *S *. *enterica* Infantis isolates from both humans and food (Table S1). Presence of pESI or a pESI-like plasmid is associated with multiple-drug resistance (Tables S1 and S4) and resistance to heavy metals in *S *. *enterica* Infantis [4, 5, 9]. Forty-two *S *. *enterica* Infantis isolates harbouring the pESI-like plasmid had *bla*_CTX-M-65_ associated with extended-spectrum β-lactam resistance. These isolates were present in a separate lineage within clade 1 (Fig. 4a). The median pairwise SNP distance between the 42 isolates within the *bla*_CTX-M-65_ clade was 34 (range 0–68) and the median pairwise SNP distance of this *bla*_CTX-M-65_ clade with the other isolates in the phylogeny was 157 (range 36–333) (Fig. 4a). Another isolate, SRR14774778, that was present in this clade did not have *bla*_CTX-M-65_. This isolate was isolated 8 months (January 2013) before the other isolates in this clade (Fig. 4b). Higher resolution of the isolates within this lineage showed multiple AMR profiles being present with AMR profile 1 (resistance to eight antimicrobial classes) found in 9/19 of the isolates (Fig. 4b, Tables S2 and S4). Based on the samples received at PHE, the first ESBL-producing human isolate seen in this study was in 2013 (Table S1). Thirteen out of the seventeen ESBL-positive human *S *. *enterica* Infantis isolates with known travel history were related to travel to South America (Fig. 4b, Tables S1 and S2). Six out of the seven poultry food products that were ESBL positive were associated with importation from South America (Fig. 4, Tables S1 and S2).

## Discussion

*S *. *enterica* Infantis has been the most prevalent serovar isolated from fresh poultry meat and broiler flocks from across Europe [6, 54] and human *S *. *enterica* Infantis infections are often associated with poultry meat [2, 6, 55–58]. Between 2014 and 2018 in England and Wales, human salmonellosis associated with *S *. *enterica* Infantis has been increasing, and this serovar has now become the fifth most widespread after *S. enterica* Enteritidis (*n*=12 548, 290.6 %), *S *. *enterica* Typhimurium including monophasic (*n*=7930, 180.7 %), *S *. *enterica* Newport (*n*=1372, 30.2 %) and *S *. *enterica* Typhi (*n*=1120, 20.6 %) (PHE data).

Of the 502 human isolates in England and Wales between 2014 and 2018 with travel information, 52.3 % were associated with travel (Table S3). Most travel-associated cases were linked to travel to Asia, followed by North America and Europe. An increasing incidence of *S *. *enterica* Infantis human cases between 2014 and 2018 was observed, with a yearly peak between July and October; this could be linked to travel-associated cases (2014, 41.4 % cases; 2015, 25.0 % cases; 2016, 26.8 % cases; 2017, 45.4 % cases; and 2018, 34.0 % cases) (Fig. 1), coinciding with the summer holidays.

The change of epidemiology and the increasing incidences of MDR clonal lineages of *S *. *enterica* Infantis isolates since the late 1990s may be related to the development of resistance to medically important antimicrobials, including extended-spectrum β-lactams [4, 5, 7, 9, 56, 59]. Infections with ESBL-producing *S *. *enterica* Infantis are particularly concerning because they are also commonly resistant to additional classes of antimicrobials [4], leaving few treatment options and the potential for poorer clinical outcomes [5].

Emergence of extended-spectrum β-lactam *bla*_CTX-M-65_ clones in England and Wales was first observed in human *S *. *enterica* Infantis isolates between 2010 and 2012 in a previous study [60], and in the current study from 2013 (Fig. 4, Table S1). *bla*_CTX-M-65_ ESBL-producing *S *. *enterica* Infantis has previously been described in Europe, and South and North America [4, 5, 10, 11, 59]. The number of *bla*_CTX-M-65_ isolates being reported in England and Wales between 2013 and 2018 increased, with 21/42 isolates reported seen in 2018 (Table S2).

Of public-health concern is the fact that in England and Wales, ESBL-producing *S *. *enterica* Infantis isolates between 2013 and 2018 were isolated from humans as well as retail poultry products imported from South America (Tables S1 and S2). As with previously reported global *S *. *enterica* Infantis isolates, phenotypic and genotypic AMR analysis also showed resistance to multiple drugs, including trimethoprim/sulfamethoxazole and ciprofloxacin, two drugs recommended for therapy of *Salmonella* infections when therapy is indicated (Table S3) [5, 9, 59]. Additionally, 28 out of the 42 isolates in the current study also contained resistance determinants for fosfomycin, an agent used to treat urinary tract infections [61] (Table S2). Recently, a clonal lineage of azithromycin-resistant *S *. *enterica* Infantis with ESBL enzymes has been reported in Peru [10]. These ESBL-producing isolates also harbour genes associated with heavy metal resistance, such as arsenic (*ars* operon) and mercury (*mer* operon) resistance (Fig. 3), as previously shown [62, 63]. Arsenic has been utilized in agriculture and poultry for antimicrobial purposes since the 1940s in the USA [62], and mercury has been utilized as medication for plant diseases and in pesticides [63]. Studies have shown co-resistance to metals and multiple antimicrobial classes in Gram-negative bacteria [64]. The co-existence of metal-resistance and AMR genes on the same genetic locus and the horizontal transfer of these co-located determinants through the *S *. *enterica* Infantis population is capable of disease requiring antimicrobial treatment and, hence, poses a threat to public health. Taken together, the results from this study and others demonstrate a highly resistant serovar that is perhaps more difficult to treat.

The emergence of MDR and ESBL-producing *S *. *enterica* Infantis has been connected with the presence of a unique pESI or pESI-like megaplasmid that enhances the fitness of the bacterium [4, 5, 9, 59]. The stability of pESI is presumed to benefit from the presence of three type II addiction (toxin–antitoxin) systems that were found in the plasmid, and such systems are believed to contribute to plasmid maintenance and prevalence during vertical transmission [65].

Genome analysis revealed the presence of a pESI-like megaplasmid of approximately 320 kbp in isolate 114061 (SRR6854755) and the presence of pESI-like plasmids in the other 368/795 *S *. *enterica* Infantis isolates studied between 2000 and 2017 (Table S1, Figs S1 and S2). pESI-like plasmids from human *S *. *enterica* Infantis isolates have been seen in the UK as early as 2000 and the first *bla*_CTX-M-65_ isolate seen in 2013 (Tables S1 and S2).

Using PlasmidFinder, we confirmed the presence of an IncFIB-like incompatibility group replicon in the complete sequences of an English human *S *. *enterica* Infantis isolate from 2015 (isolate 114061). Using brig (blast Ring Imaging Tool) and blastn, the pESI-like plasmid from 114061 (SRR6854755) shared 99 % sequence identity with a plasmid from a chicken *S *. *enterica* Infantis isolate from the USA [5] (Fig. 3). The plasmid contained genes conferring enhanced colonization capability and biofilm formation (*ipf* and *fae* fimbriae operons), virulence [fimbriae, yersiniabactin (*ybt* operon)], resistance and fitness [*tet*, *dfrA* and *sul1* genes and genes related to tolerance to heavy metals (*mer* and *ars* operons) in the intensive-farming environment, as seen in the pESI plasmid from Israel [9], with additional resistance genes *bla_CTX-M-65_* and *fosA* conferring resistance to ESBL and fosfomycin (Figs 3, S1 and S2) . The high degree of similarity between the pESI and pESI-like plasmids suggests common ancestry of these plasmids, and these plasmids are potentially acquiring different resistance determinants horizontally, most likely due to selection pressures in intensive poultry farm settings, as *S *. *enterica* Infantis is strongly associated with poultry [6, 8, 15].

Variations are often observed in the two drug regions found on pESI or pESI-like plasmids [4, 5, 59]. In this study, the 42 *bla*_CTX-M-65_ isolates from humans and food exhibited six different AMR profiles with resistance ranging from eight antimicrobial classes [including isolate 114061 (SRR6854755)] to six antimicrobial classes (Table S2). One isolate (SRR6191309) did not have the *tet(A)−1* and *sul1* genes in region 2 (AMR profile 4) that was described in the original pESI plasmid (Table S2) [9]. The variability in resistance gene compositions would be expected, since the resistance genes were surrounded by transposases and/or integrases, suggesting that they would be mobile.

Phylogenetic analysis of 795 *S *. *enterica* Infantis eBG31 isolates between 2000 and 2017, including 42 ESBL-producing isolates, was carried out to gain a broader perspective on the distribution of pESI/pESI-like plasmids and mobile genetic elements/antimicrobial properties amongst the English and Welsh *S *. *enterica* Infantis eBG31 population. A total of 368 isolates harbouring pESI-like plasmids formed a separate clade (clade 1), indicating that pESI was introduced into the English and Welsh eBG31 population prior to 2000 and clonal expansion of these isolates has occurred. Clade 1 consisted of isolates from animals, food and humans. The 42 *bla*_CTX-M-65_ isolates fell into a lineage within clade 1, 13 of the human isolates with known travel history were associated with travel to South America and 6 out of the 7 poultry products were imported from South America (Fig. 4). Recent studies have shown the wide distribution of *bla*_CTX-M-65_ in commensal bacteria in the paediatric populations in Bolivia [66], and has been described in *S *. *enterica* Infantis in the human population in Peru and Ecuador [10, 11]. The discovery of *bla*_CTX-M-65_ in pESI-like plasmids from South American *S *. *enterica* Infantis once again highlights the rapid global transmission of a highly drug-resistant pathogen through travel or the possible consumption of contaminated food products. Variations in AMR profiles are expected due to the plasticity of both the drug regions in the pESI-like plasmid and the varying sampling (in this study it’s biased towards clinical isolates that PHE receives) and sequencing times of the samples (Fig. 3). Even though the isolates were collected over a period of 17 years and from different sources, such as humans and poultry food products, the small genetic distance between the *bla*_CTX-M-65_ lineage isolates suggests a strain that likely spread by clonal expansion in the recent past and has been in circulation for some time (Fig. 4a).

The presence of pESI-like plasmids in the *S *. *enterica* Infantis population in England and Wales since 2000 and the *bla*_CTX-M-65_ ESBL isolates since 2013 has shown the emergence of a stable resistant clone that has been in circulation for some time in the human population. The limitation of the study was that 33/35 of the food isolates were poultry related but had no known country of origin (other than the 6 ESBL South American isolates) (Table S1). Therefore, this investigation does not resolve the source of introduction to either humans or animals, but does highlight how easily resistant bacteria can spread globally and suggests the possible introduction of ESBL *S *. *enterica* Infantis into England and Wales from travel or imported poultry products from South America. These findings also show the importance of using WGS data for integrated epidemiological, phylogenomics and AMR/virulence surveillance to compare animal, human and food isolates. As a result, we will be equipped not only to detect the transmission of the pathogen but also to identify different sources and mechanisms of resistance/virulence that will allow us to design more effective interventions and thorough risk-management strategies.

## Supplementary Data

Supplementary material 1

Supplementary material 2

Supplementary material 3

Supplementary material 4

Supplementary material 5

Supplementary material 6
