# Supplementary material for: Characterization of a pESI-like plasmid and analysis of multidrug-resistant Salmonella enterica Infantis isolates in England and Wales
Source: Microb Genom. 2021 Oct 14;7(10):000658. doi: 10.1099/mgen.0.000658 (PMC8627215; doi:10.1099/mgen.0.000658)
Supplement: Supplementary material 1 [file mgen-7-0658-s001.pptx]

## Slide 1
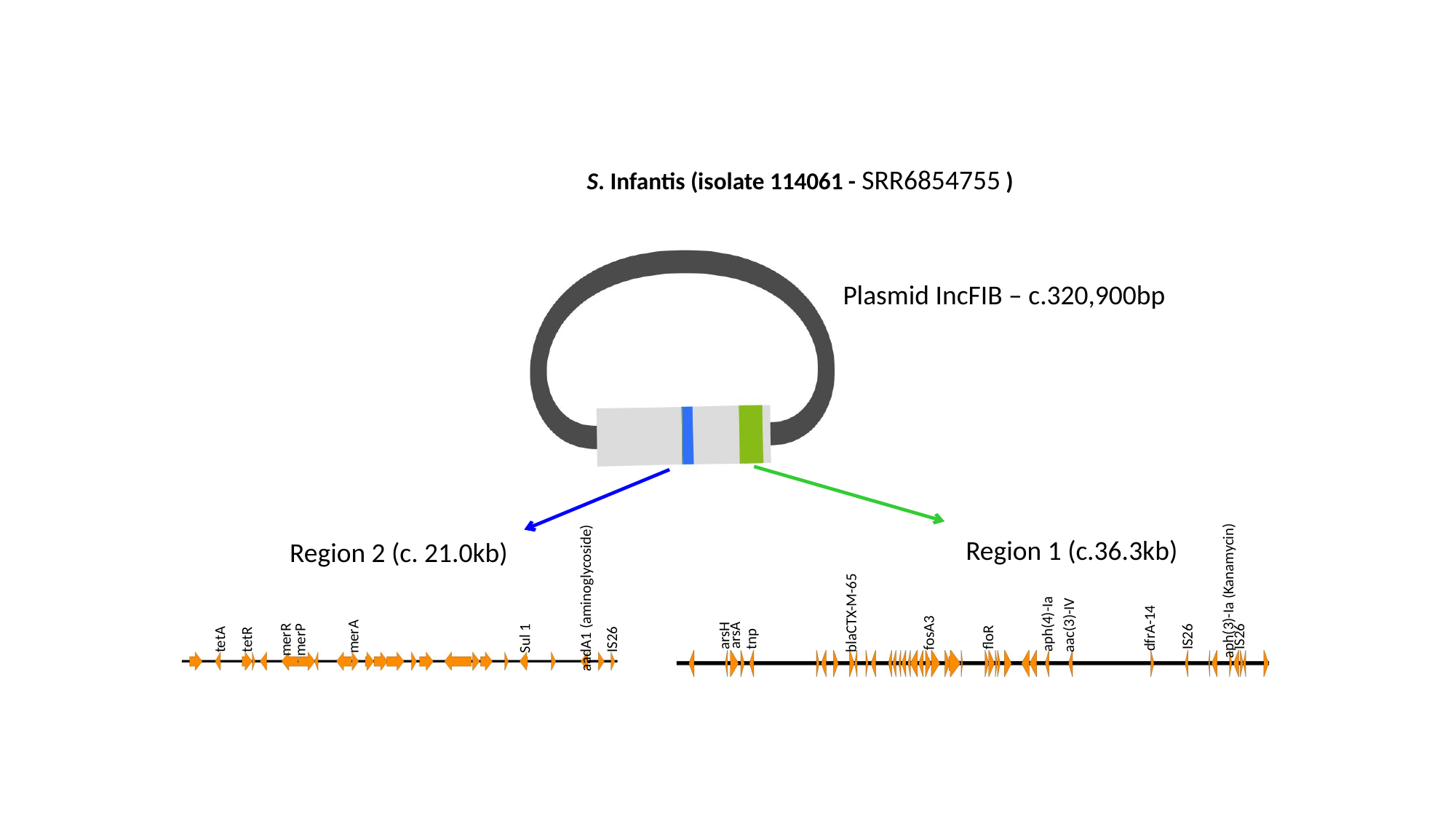

S. Infantis (isolate 114061 - SRR6854755 )
Plasmid IncFIB – c.320,900bp
aph(3)-Ia (Kanamycin)
blaCTX-M-65
aph(4)-Ia
aac(3)-IV
dfrA-14
fosA3
arsA
arsH
IS26
IS26
floR
tnp
aadA1 (aminoglycoside)
merA
Sul 1
tetA
IS26
tetR
merP
merR
Region 1 (c.36.3kb)
Region 2 (c. 21.0kb)
