# Supplementary figures and images for: Characterization of a pESI-like plasmid and analysis of multidrug-resistant Salmonella enterica Infantis isolates in England and Wales
Source: Microb Genom. 2021 Oct 14;7(10):000658. doi: 10.1099/mgen.0.000658 (PMC8627215; doi:10.1099/mgen.0.000658)

## Slide 1
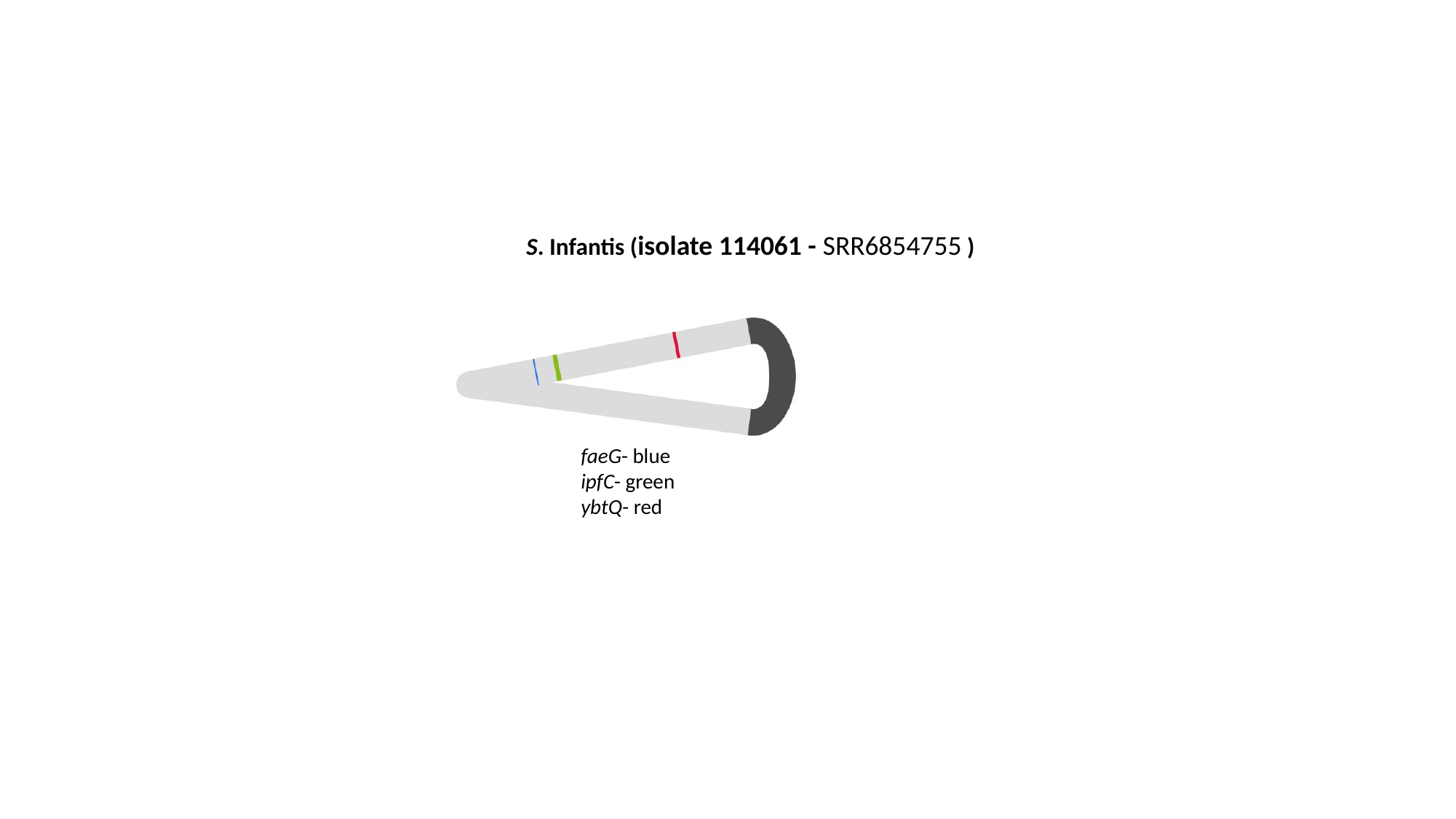

S. Infantis (isolate 114061 - SRR6854755 )
faeG- blue
ipfC- green
ybtQ- red

Supplement: Supplementary material 2 [file mgen-7-0658-s002.pptx]
